# Electric scooter accidents leading to emergency department visits: influence of alcohol and outcomes in Stockholm, Sweden

**DOI:** 10.1038/s41598-023-32857-1

**Published:** 2023-04-12

**Authors:** Olle Andersson, Therese Djärv

**Affiliations:** 1 Department of Emergency Medicine, Karolinska University Hospital, Solna, Sweden; 2 Department of Medicine Solna, Karolinska Institutet, 171 76 Stockholm, Sweden

**Keywords:** Risk factors, Trauma

## Abstract

Electric scooters are a popular form of transportation, but accidents have increased with increased usage over the last years with rising health care costs as a consequence. This study aims to quantify accidents associated with the use of alcohol and to compare injuries at the emergency department (ED) among patients who have been involved in an accident involving an electric scooter. We used data from a multi-center retrospective registry-based cohort in the Swedish Traffic Accident Data Acquisition. We included all patients that had been involved in an electric scooter accident needing a visit to all EDs in Stockholm, Sweden during 2019–2020. Data on alcohol intake was manually drawn from medical files. Out of all of the 369 patients, the majority were men (n = 223, 60%) and aged below 30 years (n = 181, 49%). In all, 102 (28%) of the patients had a positive history of alcohol intake prior to the accident. Patients with alcohol intake more often arrived nighttime than those without alcohol, via ambulance (40% and 24%, respectively, p-value < 0.01). Those with alcohol intake needed to be admitted to hospital from the ED instead of being discharged more often than those withour alcohol (17% and 9%, respectively, p-value < 0.05) even if the majority still had minor injuries. Every 33,000 trips on electric scooters are statistically accompanied by an accident leading to an ED visit. We conclude that accidents with electric scooters are often associated with alcohol intake. They often demand more resources, such as an ambulance transfer and admission to hospital, and involve multiple injuries, compared to those without alcohol intake.

## Introduction

Electric scooters are a fairly new and increasingly popular way of getting around. This has resulted in more electric scooter injuries and hospital admissions and therefore increasing costs for public healthcare^1–8^. Injuries are often minor^9,10^, but head traumas are common and severe traumas and even deaths have been reported^1,4,11–14^.

Electric scooters are also a hot topic in the public debate and some physicians have argued that accidents related to electric scooters are becoming more frequent and severe with an increased usage. Electric scooters for public rent were recently made illegal in the city of Copenhagen, Denmark^15^. Interestingly, the incidence of accidents for electric scooters is not well described and few comparison^16^ has been made to other modes of transportation.

In Sweden and other Scandinavian countries, laws and restrictions have been applied to limit the use of electric scooters. Most electric scooters are provided by companies where the driver rents the scooter and pays per minute, and when the ride ends, payment is made via a phone app. The scooter is left at the end destination point. The maximum speed for rental scooters is approximately 20 km/h. There are also a minority of electric scooters that are privately owned.

Drunk driving an electric scooter is illegal in many countries, but few studies have addressed the association between alcohol and electric scooter accidents. However, a recent study from New Zeeland showed that as many as 27% of accidents were associated with the use of alcohol^2^.

The primary aim of this study was to investigate the use of alcohol prior to an electric scooter accident and to compare the type and severity of injuries among patients with and without an accident associated with alcohol at the emergency department (ED). We also aimed to present the number of accidents in relation to electric scooter usage.

## Material and methods

### Study design, data source and study population

For this retrospective cohort study we used data from The Swedish Traffic Accident Data Acquisition (STRADA)^17^ to find all adult (at least 18 years) patients that had been involved in an electric scooter accident and who needed to visit an ED during the period 2019–2020 in Stockholm, Sweden. The STRADA contains information on individual road traffic injuries and accidents from the entire Swedish national road transport system^7^. All types of accidents, including those involving pedestrians, bicycles, and trains/trams, are reported in the registry by the Swedish police. All accidents generating a hospital visit are reported by hospitals nationwide. The reported information includes geographical location, severity and cause of the accident, type of accident, and degree of personal and property damage.

The police in Sweden report all traffic accidents in which a person is injured to STRADA. The hospitals also report all traffic accidents in which a person is injured and needs emergency medical care to STRADA. All methods were carried out in accordance with the Helsinki declaration and informed consent for inclusion in STRADA and research based on it was provided from particpants. The Regional Ethical Review Board in Stockholm, Sweden approved the study (Ref. no. 2020-04186). No patient and public involvement was done.

### Data collection and categorization

Patient and event data such as; sex, age (categorized in 5–10-year intervals), whether or not the person was wearing a helmet (yes/no/missing), time of the day (categorized in 6-h intervals), weekday and month (categorized in 3-month intervals) were collected from STRADA.

Data about the accident were also gathered from STRADA, such as arrival via Emergency Medical Services (EMS) to the ED (yes/no), whether the patient was discharged from the ED or admitted to the hospital, the severity of the injuries if multiple (AIS categorized as 1/2/3/missing, ISS categorized as none/1–3/4–8/at least 9/missing), number of injuries (collected as continuous without any upper limit and then categorized as 1/2/3–5/at least 6), type of injuries (categorized as cervical spine/concussive injury/external/internal organs/joints/muscle tendons, ligaments/skeletal/whole area with possibilities to add free text to describe the injury).

Without having any information about the injuries, we used the Swedish personal identification number^18^ to access the patient’s electronic medical file via a system called Take-Care^®^.

Information on alcohol use was accessed by manually screening the medical file for written documentation by the physician in charge regarding any use of alcohol prior to the accident. The data on alcohol in the medical file could be based on self-reported use of alcohol by the patient or a positive s-ethanol blood sample or a positive breath alcohol test. It is not mandatory to document alcohol usage before an accident in the ED but usual practice among doctors to do so in our settings. Data on alcohol was categorized as yes/no, if data on alcohol use were missing we categorized the patient as no.

Data regarding the total amount of journeys, kilometres driven, active time on the electric scooter were based on reported data from the four companies active in Stockholm during the period 2019–2020. Data were collected directly from each of the four companies by a third party (the Micromobility Association, including all the major electric scooter companies in Stockholm), and presented anonymized for each company.

### Statistical analysis

Descriptive statistics were used for demographic variables. Differences between injuries among patients with alcohol intake and those without alcohol intake were tested for statistical significance across groups with Chi-squared test on the significance level of 0.05. All statistical analyses were conducted using STATA 10.2 TX software.

## Results

### Characteristics of patients in relation to the use of electric scooters

**Figure 1:**
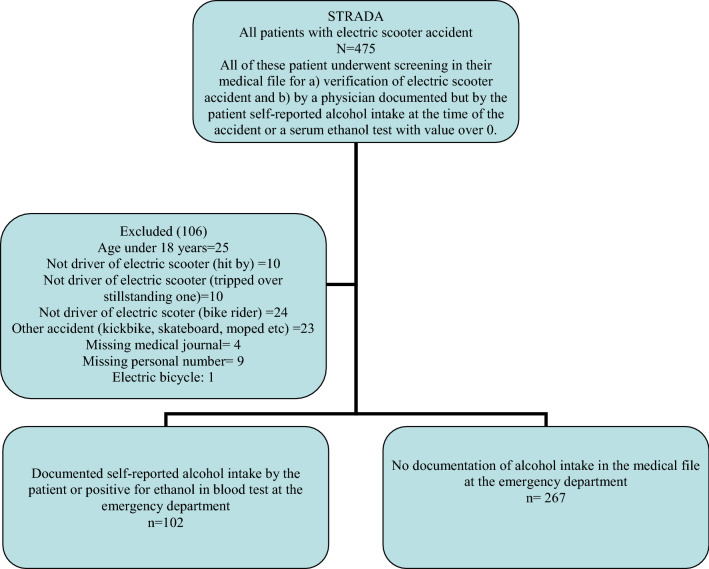
Flowchart for study population.

In all, STRADA included 475 patients, of which 106 fulfilled the criteria for exclusion (Fig. 1). Out of the remaining 369 patients, regardless of alcohol use or not, the majority were men (n = 223, 60%) and aged below 30 years (49%). Helmets were only used by every tenth patient and two-thirds of injured patients arrived to the ED during evenings and nighttime, i.e. between 18.00 and 06.00. Every fourth injured patient arrived to the ED on a Saturday and half of the injuries occurred during the summer (Table 1).

**Table 1:** Characteristics of all patients visiting an emergency department due to an accident with an electric scooter in Stockholm, Sweden 2019–2020.

	Patient with alcohol* associated with the accident Number (%) 102 (100)	Patient without alcohol* associated to the accident Number (%) 267 (100)	p-value**
Gender			
Female	36 (35)	110 (41)	0.30
Male	66 (65)	157 (59)	
Age categories (years)			
18–24	37 (36)	64 (24)	0.35
25–29	16 (16)	64 (21)	
30–34	16 (16)	32 (12)	
35–39	11 (11)	36 (13)	
40–44	8 (8)	15 (6)	
45–49	5 (5)	19 (7)	
50–59	8 (8)	24 (9)	
60–69	1 (1)	7 (3)	
At least 70	0 (0)	1 (1)	
Wearing a helmet			< 0.01
Yes	3 (3)	35 (13)	
Missing	19 (19)	31 (12)	
Time of the day			
00.00–06.00	69 (70)	66 (25)	< 0.01
06.01–11.59	1 (1)	35 (13)	
12.00–17.59	1 (1)	74 (28)	
18.00–23.59	28 (28)	86 (33)	
Missing	3 (3)	6 (2)	
Weekday			< 0.01
Monday	3 (3)	27 (10)	
Tuesday	5 (5)	34 (13)	
Wednesday	8 (8)	35 (13)	
Thursday	22 (22)	31 (12)	
Friday	15 (15)	51 (19)	
Saturday	32 (31)	57 (21)	
Sunday	17 (16)	32 (12)	
Time of the year			0.02
Winter (December–February)	10 (9)	7 (3)	
Spring (March–May)	7 (7)	29 (11)	
Summer (June–August)	52 (51)	133 (50)	
Autumn (September–November)	33 (32)	98 (37)	

*Alcohol in blood was determined as either a note of self-reported intake of alcohol in the medical file written by the physician or a value over 0 on the laboratory test serum-ethanol taken at arrival to the emergency department.

**Chi squared test between all patients with alcohol compared to those without alcohol.

### Difference between drivers with and without documented alcohol intake

Of 369 patients, 102 (28%) patients had been involved in an accident associated with a positive history of alcohol use. In 8 patients, positive serum ethanol was available, ranging from 29 to 66 mmol/L corresponding to 1.09–2.48‰. Further, 21 patients had a positive breath alcohol test ranging from 0.4 to 2.21‰. The remaining 71 patients had a self-reported intake of alcohol documented in the medical file by the physician in charge. A negative serum ethanol blood sample was available in 2 patients only. Alcohol history was found in almost all age categories but most often in younger patients (Table 1). The use of helmets was very rare, and even more rare among those with alcohol intake (3% and 13%, respectively, p-value < 0.01). Two-thirds of injured patients with alcohol history arrived to the ED between midnight and 06.00, every third arrived on a Saturday during the summer period.

Patients with a positive history of alcohol use more often arrived via EMS and needed to be admitted to a hospital from the ED instead of being discharged, even if the majority still had minor injuries (Table 2). Further, they more often had multiple injuries and concussive injuries.

**Table 2:** Injury pattern of all patients visiting an emergency department due to an accident with an electric scooter in Stockholm, Sweden 2019–2020.

	Patient with alcohol* Number (%) 102 (100)	Patient without alcohol Number (%) 267 (100)	p-value**
Arrival mode to ED			
Emergency Medical Services	41 (40)	24 (9)	< 0.01
Admission in the ED			< 0.01
Left without seen	3 (3)	1 (< 1)	
Discharged	82 (80)	241 (90)	
Admitted to hospital	17 (17)	25 (9)	
Accident degree (ISS)			
None	4 (4)	32 (12)	< 0.01
Minor (ISS 1–3)	63 (62)	138 (52)	
Intermediate (ISS 4–8)	27 (26)	93 (35)	
Serious (ISS 9–)	8 (8)	3 (1)	
Missing	0 (0)	1 (< 1)	
AIS-degree			0.04
0	3 (3)	24 (9)	
1	63 (62)	138 (53)	
2	31 (31)	93 (36)	
3	4 (4)	3 (1)	
4–6	0 (0)	2 (1)	
Missing	1 (1)	7 (2)	
Number of injuries reported			< 0.01
1	43 (42)	187 (70)	
2	26 (25)	43 (16)	
3–5	30 (29)	37 (14)	
6–13	3 (3)	1 (< 1)	
Type of injury***			
Cervical spine	3 (3)	3 (1)	0.35
Concussive injury	20 (20)	14 (5)	< 0.01
External (mainly hands and face)	7 (7)	7 (3)	0.06
Internal organs	6 (6)	1 (< 1)	< 0.01
Joints (mainly foots and knees)	10 (10)	22 (8)	0.63
Muscle tendons, ligaments	0 (0)	10 (4)	0.07
Skeletal	42 (41)	109 (41)	0.93
Whole area (mainly face, hands, knees and foots)	60 (59)	119 (44)	0.01

*Alcohol in blood was determined as either a note of self-reported intake of alcohol in the medical file written by the physician or a value over 0 on the laboratory test serum-ethanol taken at arrival to the emergency department.

**Chi squared test or Fischers 2-sided exact test if less than 5 per cell.

***Type of injury, all injuries without any upper limit of accidents per patient could be reported.

### Usage in relation to accidents

The total number of journeys in 2019 and 2020 in Stockholm was 11,551,922; the total travel distance was 16,989,608 km; the total time spent on an electric scooter was 1,599,967 h. The total amount of adult accidents needing an ED visit was 377. In other words, the incidence of an accident on an electric scooter that needed emergency medical care during the period 2019–2020 in Stockholm was approximately 1:31,000 journeys, or 1:45,500 km, or 1:4,300 h.

## Discussion

To our knowledge, this is one of the larger population-based cohorts based on both police and hospital reported accidents in the field of electric scooter accidents, and one of few assessing the influence of alcohol. Our main findings are that 28% of accidents were associated with alcohol intake, drivers under the influence of alcohol more often had multiple injuries.

The percentage of accidents associated with alcohol varies in the literature between 4 and 27%^2,11,19,20^, this might relate partly to different cultures and laws in different settings as well as due to study designs. Our percentage being one of the highest might relate to our manual screening of the medical charts for self-reported intake and not only laboratory tests. On the other hand, if we had used routine tests in clinical practice, our ratio might have been even higher. For example, Kobayashi et al.^21^ used blood samples and found levels above the legal limit in about half of the patients.

This is one of few studies, to our knowledge, where the incidence of accidents on electric scooters is presented. Our incidence rate (3 per 100,000 trips, or 20:1,000,000 km, or 23 per 100,000 h) is much lower than in a recent study from New Zealand presenting 20 per 100,000 trips^2^. The differences could at least partly be explained by the fact that their number was an estimate and ours were based on exact figures. Interstingly, a recent study from Norway have presented a threefold incidence of accidents among scooters compared to bikes^16^. This can also be compared with accident rates for ED visits for bicycles from a prospective cohort study from Belgium^22^; 3 per 100,000 trips, 5 per 1,000,000 km and 8 per 100,000 h. Therefore, it seems that electric scooter drivers travel a shorter distance and a shorter time before an accident occurs. Of note, we only collected the data from rental companies since private-owned electric scooters were rare at the time of data collection.

Regarding patient and usage characteristics, we found similar characteristics in previous studies, i.e. most riders are men and young, and usage is related to night times and weekends^2,11,19,20^.

The finding of more multiple injuries and concussion injuries among alcohol users deserves attention. The use of a helmet was equally or even more rarely used in our cohort compared to previous studies, ranging from 1 to 19%^11,20,21,23–26^. For now, there is no legal retrictions on helmet and electric scooters in Sweden and it remains unknown whether the use of a helmet would be effective in preventing these injuries even if previous studies have shown that traumatic brain injuries that result from bicycle and skateboard accidents decrease significantly with the use of a helmet^27,28^. Regarding other types of injuries, our second most common injury was skeletal/fractures and this corresponds quite well with other studies^2,11,19–21^.

A limitation in our study was the lack of consistency in screening for alcohol in the hospitals in Stockholm. When we take into consideration the fact that most of the accidents occurred on weekends between 18:00 and 06.00, in the central part of Stockholm and that most of the patients were 30 years of age or less, and that it is not mandatory to assess or document alcohol usage when involved in an accident with an electric scooter, it is natural to suspect that the use of alcohol prior to an accident on an electric scooter is underreported rather than the opposite. Further, our dataset is to small to run regression analysis on severity type and adjust for helmet use and so on.

A strength is that we used population-based mandatory registration with high completeness as our main data source. The STRADA database collects both police-reported accidents and hospital reported accidents which cover all accidents rather than only a minor portion of them. From STRADA we know that the total numbers of electric scooter accidents were higher since we only included accidents by the driver of the electric scooter and those needing a visit to the ED.

Future interventional studies aiming for a reduction of the number of accidents, especially associated with alcohol, might look into the possibility of pausing the rental of electric scooters during the night or in other ways making sure that the driver is not under the influence of alcohol. In Sweden, it is already illegal to drive a vehicle under the influence of alcohol, including electric scooters.

In conclusion, accidents with electric scooters are often associated with alcohol intake. Every 33,000 trips on an electric scooter statistically involve an accident that requires a visit to an ED.

## Data Availability

Please contact corresponding author, therese.djarv@ki.se for data requests.

## Abbreviations

AIS: Abbreviated injury scale
ED: Emergency department
EMS: Emergency medical services
ISS: Injury Severity Score
STRADA: The Swedish Traffic Accident Data Acquisition
